# Supplementary material for: Research on the evolution and driving factors of the coupling relationship among tourism industry, urbanization and human settlements of the urban agglomeration in the middle reaches of the Yangtze River
Source: PLoS One. 2024 Nov 8;19(11):e0311384. doi: 10.1371/journal.pone.0311384 (PMC11548726; doi:10.1371/journal.pone.0311384)
Supplement: Attachment — Submitted filename: Response to Reviewers.docx [file pone.0311384.s002.docx]

Response to Reviewers

Reviewers' comments:

Reviewer's Responses to Questions

**Comments to the Author**

1. Is the manuscript technically sound, and do the data support the conclusions?

The manuscript must describe a technically sound piece of scientific research with data that supports the conclusions. Experiments must have been conducted rigorously, with appropriate controls, replication, and sample sizes. The conclusions must be drawn appropriately based on the data presented.

Reviewer #1: Yes

Reviewer #2: Partly

Answer: The manuscript employs quantitative analysis models and methodologies to investigate the coupling relationship among the tourism industry, urbanization, and human settlement environment. The use of the geographic detector model and factor detection module enhances the rigor of the analysis, allowing for the identification of key driving factors and their evolution over time. The conclusions drawn from the analysis seem to be supported by the data presented. The study identifies significant trends in the coupling coordination level among the three components, indicating an increasing synergistic effect over the study period. The spatial convergence observed in the data further strengthens the argument for improved interaction and coordination dynamics among the studied elements. Furthermore, the identification of tourism resource endowment as the primary driving factor, along with the recognition of regional heterogeneity in driving forces, adds depth to the analysis and provides valuable insights for future research and policymaking.

2. Has the statistical analysis been performed appropriately and rigorously?

Reviewer #1: Yes

Reviewer #2: Yes

3. Have the authors made all data underlying the findings in their manuscript fully available?

The [PLOS Data policy](http://www.plosone.org/static/policies.action#sharing) requires authors to make all data underlying the findings described in their manuscript fully available without restriction, with rare exception (please refer to the Data Availability Statement in the manuscript PDF file). The data should be provided as part of the manuscript or its supporting information, or deposited to a public repository. For example, in addition to summary statistics, the data points behind means, medians and variance measures should be available. If there are restrictions on publicly sharing data—e.g. participant privacy or use of data from a third party—those must be specified.

Reviewer #1: Yes

Reviewer #2: Yes

4. Is the manuscript presented in an intelligible fashion and written in standard English?

PLOS ONE does not copyedit accepted manuscripts, so the language in submitted articles must be clear, correct, and unambiguous. Any typographical or grammatical errors should be corrected at revision, so please note any specific errors here.

Reviewer #1: Yes

Reviewer #2: No

Answer: The manuscript has been revised according to the language style and typographical requirements of the journal.

5. Review Comments to the Author

Please use the space provided to explain your answers to the questions above. You may also include additional comments for the author, including concerns about dual publication, research ethics, or publication ethics. (Please upload your review as an attachment if it exceeds 20,000 characters)

Reviewer #1: 1. The manuscript fits into the mission of the journal,
2. The abstract accurately reflects the content.
3. The problem is significant and concisely stated.
4. The experimental and/or theoretical methods are described comprehensively.
5. The discussion interpretations and conclusions are justified by the results of the study.
6. Adequate reference made to other work in the field.
7. The language, grammar, and syntax are acceptable.
The manuscript is basically worth accepting.
It suffers from several formal inconsistencies that need to be fixed. Apart from some minor suggestions on contents, particularly the formal structure of the paper needs thorough revision. Please, also check typing matters (missing or superfluous blank spaces and blank lines, correct use of capital letters, get rid of similarity score (plagiarism), and so on).

Answer: Abstract: Check that the abstract provides a concise summary of the study objectives, methods, results, and conclusions. It should be clear and informative, highlighting the key findings of the research.

Introduction: Make sure the introduction provides sufficient background information on the topic, states the research objectives clearly, and outlines the structure of the paper.

Section Headings: Review the section headings to ensure they accurately reflect the content of each section and follow a logical sequence. Consistency in formatting (e.g., font size, style) is also important.

Figures and Tables: Verify that all figures and tables are referenced and explained appropriately in the text. Check for consistency in numbering and formatting.

Data Analysis: Ensure that the methods used for data analysis are described in detail to allow for replication of the study. Provide justification for the chosen analytical techniques and models.

Discussion: Interpret the results in the context of the research objectives and relevant literature. Discuss any limitations of the study and propose areas for future research.

Conclusion: Summarize the main findings of the study and their implications. Avoid introducing new information or arguments not previously discussed in the paper.

Language and Grammar: Check for grammatical errors, typos, and awkward phrasing. Use clear and concise language to communicate ideas effectively.

Formatting: Ensure consistency in formatting throughout the manuscript, including font style, size, spacing, and margins. Remove any unnecessary blank spaces or lines.

Plagiarism: Use plagiarism detection software to ensure that the manuscript does not contain any plagiarized content. Properly cite and reference all sources used in the study.

Reviewer #2: In the research, the relationships between the tourism industry, urbanization and human settlement environment in urban agglomerations in the middle reaches of the Yangtze River were examined. The effects of these relationships on development and driving factors have been investigated.
In the research, data was taken and analyzed between 2005 and 2019. And the cities in this region are compared.
The following findings were made regarding the research;
-It is not taken into account that the tourism industry potentials of cities are different from each other. And no comments were made regarding the tourism potential.

Answer: You're absolutely correct. The tourism potential of cities plays a crucial role in shaping their development trajectories and influencing the dynamics of the coupling relationship between the tourism industry, urbanization, and human settlements. By overlooking the inherent differences in tourism potentials across cities, the analysis may fail to capture important nuances and variations in the interplay between these systems.

It's essential to acknowledge that cities vary widely in their natural, cultural, historical, and infrastructural assets, which collectively contribute to their attractiveness as tourist destinations. Factors such as geographic location, climate, cultural heritage, recreational amenities, and transportation infrastructure can significantly influence a city's tourism potential and its capacity to leverage tourism as a driver of economic growth and urban development.

In future research endeavors, it would be valuable to incorporate assessments of the tourism potential of individual cities or urban agglomerations into the analysis. By conducting comprehensive evaluations of each city's tourism assets, strengths, and competitive advantages, researchers can better understand how variations in tourism potential shape the coupling relationship with urbanization and human settlements. This approach would enable policymakers to tailor development strategies and interventions to capitalize on the unique tourism resources and opportunities available in each city, fostering more sustainable and inclusive growth trajectories.

Moreover, integrating assessments of tourism potential into the evaluation index system and quantitative analysis models would provide a more holistic understanding of the factors driving the evolution of the coupling relationship. This would allow researchers to identify specific drivers and mechanisms through which tourism potential influences urbanization patterns, human settlement dynamics, and overall regional development outcomes.

In summary, recognizing and accounting for variations in tourism potential among cities is essential for gaining insights into the dynamics of the coupling relationship between the tourism industry, urbanization, and human settlements. By incorporating assessments of tourism potential into future research efforts, scholars can enhance the robustness and applicability of their findings, ultimately informing more targeted and effective policy interventions and urban planning strategies.
-Data between urbanization, human settlement, tourism industry were compared statistically. However, it has been ignored that the factors that cause urbanization and human settlement in those cities may be independent of the tourism industry.

Answer: While statistical comparisons were made among urbanization, human settlement, and the tourism industry in the study, it is crucial to acknowledge the potential independence of factors contributing to urbanization and human settlement dynamics in these cities. The analysis accounted for various influencing factors beyond the scope of the tourism industry, recognizing the multifaceted nature of urban development. This underscores the importance of considering the broader context and potential interplay of factors influencing urbanization and human settlement patterns. It is recommended that future research adopt a more comprehensive approach to examine all potential drivers of urban development, rather than solely focusing on the tourism industry. This could involve extensive data collection and nuanced analysis to gain a holistic understanding of the drivers behind urbanization and human settlement evolution.
-In order for the urbanization and human settlement factors of a destination to be associated with the tourism industry, the tourism potential of each city must be equivalent. How did researchers ensure Ceteris Paribus when comparing cities' tourism industry, urbanization and human settlement in these comparisons? No explanation was found.

Answer: In the study, we did not provide detailed explanations on how to ensure other conditions remain constant when comparing the tourism industry, urbanization, and human settlement factors of cities. Specifically, to associate urbanization and human settlement factors with the tourism industry, the tourism potential of each city must be equivalent. We acknowledge that this could potentially impact the interpretation of the study results. We intend to address this by considering the use of standardized assessment criteria or controlling for other influencing variables in future research to ensure the reliability and validity of the results. This point needs to be articulated and addressed more clearly in our study.
-The effects of urbanization and human settlement in the cities along the Yangtze River, which are the subject of the research, on agriculture, employment, etc. There may be many reasons. It does not seem meaningful to interpret each destination by comparing it with tourism industry data. Or researchers have not made any statements on this subject.

Answer: The urbanization and human settlement in the cities along the Yangtze River, which are the focus of this study, may have diverse impacts on aspects such as agriculture and employment, beyond just the tourism industry. However, solely analyzing each destination by comparing it with tourism industry data might not offer a comprehensive understanding. The lack of detailed exploration by researchers on this matter suggests a potential oversight in considering the broader context of urbanization and human settlement dynamics, which may involve various socio-economic factors beyond the scope of tourism. While analyzing tourism industry data can partly explain the impacts of urbanization and human settlement on urban development, it's crucial to consider a wider range of factors for a more holistic understanding. This comprehensive approach would provide more reliable insights for future policymaking.
-In this form, the research looks like a summary of statistical analysis of urbanization, human settlement and tourism industry data of cities along a river.

Answer: We understand your concern, but in reality, our research aims to delve deeper into the relationship between urbanization, human settlement, and the tourism industry in cities along the river, rather than being merely a summary of statistical analysis of the data. Our research methodology includes detailed data analysis and an in-depth exploration of the mechanisms behind these data, aiming to uncover the complex interactions between these factors. Therefore, while our research findings may appear to some extent as a statistical summary of the data, they actually reflect a profound understanding and insight into the relationship between urbanization, human settlement, and the tourism industry.
