# Supplementary material for: Research on the evolution and driving factors of the coupling relationship among tourism industry, urbanization and human settlements of the urban agglomeration in the middle reaches of the Yangtze River
Source: PLoS One. 2024 Nov 8;19(11):e0311384. doi: 10.1371/journal.pone.0311384 (PMC11548726; doi:10.1371/journal.pone.0311384)
Supplement: Attachment — Submitted filename: Response to Reviewers0802.docx [file pone.0311384.s003.docx]

**Comments to the Author**

1. If the authors have adequately addressed your comments raised in a previous round of review and you feel that this manuscript is now acceptable for publication, you may indicate that here to bypass the “Comments to the Author” section, enter your conflict of interest statement in the “Confidential to Editor” section, and submit your "Accept" recommendation.

Reviewer #2: All comments have been addressed

Reviewer #3: (No Response)

2. Is the manuscript technically sound, and do the data support the conclusions?

The manuscript must describe a technically sound piece of scientific research with data that supports the conclusions. Experiments must have been conducted rigorously, with appropriate controls, replication, and sample sizes. The conclusions must be drawn appropriately based on the data presented.

Reviewer #2: Yes

Reviewer #3: Yes

3. Has the statistical analysis been performed appropriately and rigorously?

Reviewer #2: Yes

Reviewer #3: Yes

4. Have the authors made all data underlying the findings in their manuscript fully available?

The [PLOS Data policy](http://www.plosone.org/static/policies.action#sharing) requires authors to make all data underlying the findings described in their manuscript fully available without restriction, with rare exception (please refer to the Data Availability Statement in the manuscript PDF file). The data should be provided as part of the manuscript or its supporting information, or deposited to a public repository. For example, in addition to summary statistics, the data points behind means, medians and variance measures should be available. If there are restrictions on publicly sharing data—e.g. participant privacy or use of data from a third party—those must be specified.

Reviewer #2: Yes

Reviewer #3: Yes

5. Is the manuscript presented in an intelligible fashion and written in standard English?

PLOS ONE does not copyedit accepted manuscripts, so the language in submitted articles must be clear, correct, and unambiguous. Any typographical or grammatical errors should be corrected at revision, so please note any specific errors here.

Reviewer #2: Yes

Reviewer #3: No

Answer: The manuscript has been revised according to the language style and typographical requirements of the journal.

6. Review Comments to the Author

Please use the space provided to explain your answers to the questions above. You may also include additional comments for the author, including concerns about dual publication, research ethics, or publication ethics. (Please upload your review as an attachment if it exceeds 20,000 characters)

Reviewer #2: Thank you for your thoughtful response to my entire review report.
There are simple spelling errors in some parts of the manual. I suggest you reconsider.
Good luck

Reviewer #3: On the basis of investigating the coupling mechanism of tourism industry, urbanization and human settlement environment in the urban agglomerations of the middle reaches of the Yangtze River, this paper establishes a coupling evaluation index system of tourism industry, urbanization and human settlement environment, by means of entropy method, coupling coordination degree, coefficient of variation, Taylor index and geographical detector, this paper analyzes the general evolution trend, evolution characteristics and driving factors of the coupling relationship among tourism industry, urbanization and human settlement environment in the urban agglomerations of the middle reaches of the Yangtze River from 2005 to 2019. The overall structure of the article is complete, the workload is sufficient, but based on the following reasons, do not recommend the publication of this article:
First, the Format issues. (1) errors in references, such as the repetition of references [30] and [31]; (2) the inconsistency of line spacing in part of the formula; (3) section 4.2 does not exist in chapter 4, so there is no need for a separate sub-heading-4.1 factor test results.
Second, the problem of language expression. (1) writing error, the toponym of the first conclusion in the abstract is not consistent with the toponym of the first conclusion in chapter 5; (2) basic syntax problems, such as an incorrect use of between in the title, should use among; 2.1.4 Nm and N after the verb form to use errors, should not use three singular, etc. .
Third, the content of research. (1) in Chapter 1, whether the interaction between the two has negative effects needs to be further explored; (2) The regional profile of the study needs to be further supplemented, it introduces which cities were originally included in the middle reaches of the Yangtze River, and then breaks down into three urban agglomerations by the Taylor Index; (3) the conclusion and discussion of chapter 5 did not elaborate on the results of the discussion. It is suggested that this chapter be further divided into conclusions, discussions and implications, and research prospects.

Answer: 1. The manuscript has been rechecked for references, and citations have been updated.

2. Formatting errors have been checked and corrected individually.

3. Basic grammatical errors have been corrected.

4. The phrase "whether the interaction between the two has negative effects needs to be further explored" has been added to Chapter 1, along with an explanation.

Additionally, whether the interaction between the tourism industry and urbanization has negative effects needs to be further explored. While existing research highlights the positive correlation and mutual benefits, potential adverse impacts such as environmental degradation, social displacement, and infrastructure strain have not been thoroughly examined. This study aims to address this gap by investigating both the positive and negative consequences of this interplay, thereby providing a more balanced and comprehensive understanding of the dynamics at play.

5. The regional profile of the study has already been further supplemented.

The regional profile of the study needs to be further supplemented. It introduces which cities were originally included in the middle reaches of the Yangtze River, and then breaks down into three urban agglomerations by the Taylor Index. The cities included in the middle reaches of the Yangtze River are Wuhan, Changsha, Nanchang, Yueyang, Zhuzhou, Xiangtan, Yichang, and Jiujiang. Using the Taylor Index, these cities are further categorized into three urban agglomerations: the Wuhan metropolitan area, the Changsha-Zhuzhou-Xiangtan urban agglomeration, and the Poyang Lake urban agglomeration. This classification allows for a more nuanced analysis of the coupling coordination degree and its evolution over time, highlighting the spatial heterogeneity within the broader region.

6. The chapters have already been further divided into conclusions, discussions and implications, and research prospects.

7. PLOS authors have the option to publish the peer review history of their article ([what does this mean?](https://journals.plos.org/plosone/s/editorial-and-peer-review-process#loc-peer-review-history)). If published, this will include your full peer review and any attached files.


If you choose “no”, your identity will remain anonymous but your review may still be made public.


**Do you want your identity to be public for this peer review?** For information about this choice, including consent withdrawal, please see our [Privacy Policy](https://www.plos.org/privacy-policy).

Reviewer #2: No

Reviewer #3: No
