# Supplementary material for: Research on the evolution and driving factors of the coupling relationship among tourism industry, urbanization and human settlements of the urban agglomeration in the middle reaches of the Yangtze River
Source: PLoS One. 2024 Nov 8;19(11):e0311384. doi: 10.1371/journal.pone.0311384 (PMC11548726; doi:10.1371/journal.pone.0311384)
Supplement: Attachment — Submitted filename: Response to Reviewers0825.docx [file pone.0311384.s004.docx]

Modification description

Dear academic editor:

Based on the feedback from you and reviewers, the authors have carefully revised the paper, and the specific modifications are as follows:

1. The authors have redrawn all the figures in the paper, including Fig1，Fig 2，Fig 3 and Fig 4，and deleted Figure 5 because the content expressed in Figure 5 was repeated with that in Table 3.

2. The authors carefully examined the data in the paper, all the data have clear sources and explanations, and the authors have submitted the original data table in the last round of revision.

3. The authors have carefully read through the paper and modified the relevant text.

4. The authors have carefully reviewed the references in the paper, and they are all complete and correct.

Thank you for your hard work.

Kind regards

Ailiang Xie
